# Musculoskeletal pain and symptoms in pregnancy: a descriptive study

**DOI:** 10.1177/1759720X18812449

**Published:** 2018-11-19

**Authors:** Serdar Kesikburun, Ümüt Güzelküçük, Ulaş Fidan, Yasin Demir, Ali Ergün, Arif Kenan Tan

**Affiliations:** Department of Physical Medicine and Rehabilitation, University of Health Sciences, Gülhane School of Medicine, Gaziler Physical Therapy and Rehabilitation Research and Training Hospital, Gaziler Fizik Tedavi ve Rehabilitasyon Eğitim Araştırma Hastanesi 06800 Bilkent-Ankara, Turkey; Department of Physical Medicine and Rehabilitation, University of Health Sciences, Gülhane School of Medicine, Gaziler Physical Therapy and Rehabilitation Research and Training Hospital, Ankara, Turkey; Department of Gynecology and Obstetrics, University of Health Sciences, Gulhane School of Medicine, Ankara, Turkey; Department of Physical Medicine and Rehabilitation, University of Health Sciences, Gülhane School of Medicine, Gaziler Physical Therapy and Rehabilitation Research and Training Hospital, Ankara, Turkey; Department of Gynecology and Obstetrics, University of Health Sciences, Gulhane School of Medicine, Ankara, Turkey; Department of Physical Medicine and Rehabilitation, University of Health Sciences, Gülhane School of Medicine, Gaziler Physical Therapy and Rehabilitation Research and Training Hospital, Ankara, Turkey

**Keywords:** female, musculoskeletal problems, pregnancy

## Abstract

**Background:**

Pregnancy-induced hormonal and physiologic changes increase the risk of musculoskeletal problems in pregnancy. The purpose of this report is to provide a comprehensive look at the musculoskeletal pain and symptoms experienced during pregnancy.

**Methods:**

A total of 184 women (mean age 30.9 ± 5.0 years) who gave birth in the obstetrics clinic of a tertiary hospital were included in the study. The participants who had given birth at 37–42 weeks of pregnancy (term pregnancy) and aged over 18 years were selected for participation. Basic demographic and clinical characteristics of the participants including age, body mass index, weight gained during pregnancy, education level, occupation, parity, sex of baby, and exercise habits were collected from the medical chart and face-to-face interviews. Musculoskeletal pain sites were defined as hand–wrist, elbow, shoulder, neck, back, low back, hip, knee, and ankle–foot in a diagram of the human body. The interviews with participants were performed to assess their musculoskeletal pain separately at each trimester follow-up visit.

**Results:**

The most frequent musculoskeletal complaints during pregnancy were low back pain (*n* = 130, 70.7%), back pain (*n* = 80, 43.5%), hand–wrist (*n* = 61, 33.2%) and hip pain (*n* = 59, 32.1%). The participants experienced musculoskeletal pain most in the third trimester except for elbow, shoulder and neck pain compared with the first and second trimesters (*p* < 0.05).

**Conclusions:**

The results of the study suggest that numerous musculoskeletal problems may complicate pregnancy especially in the third trimester.

## Introduction

Pregnancy-induced biomechanical, hormonal, and vascular changes are likely to give rise to a wide variety of musculoskeletal problems.^1^ The enlarging uterus alters body’s center of gravity and applies mechanical stress on the body.^2^ Joint laxity develops secondary to hormone level fluctuations. Fluid retention leads to compression of soft tissues in pregnancy. Consequently, a pregnant woman is susceptible to musculoskeletal injuries. It has been suggested that almost all women complain of musculoskeletal problems to some extent. A one quarter of the pregnant women experience at least temporarily disabling symptoms during pregnancy.^3^ Spinal pain has been reported as the most frequent disorder.^4^ Other common problems consist of lower and upper extremity pain, muscle cramps and peripheral neuropathies.^3,4^

Musculoskeletal problems have been shown as a major source of disability and loss of work among pregnant women.^5,6^ Despite the importance of the topic, current literature is scarce for clinical trials and most reports are based on case series and expert opinion from clinical experience.^7^ The purpose of this study was to provide a comprehensive look at the musculoskeletal problems experienced during pregnancy and to document the frequencies, clinical characteristics and progressions through pregnancy with a clinical observational study.

## Patients and methods

### Study design and participants

The study was conducted from October 2013 to September 2015 at the Department of Obstetrics and Gynecology, Gülhane School of Medicine, University of Health Sciences, Ankara, Turkey. A total of 184 women were included in the study. All successive pregnant women were enrolled. Antenatal and postnatal follow up of the participants were conducted in the same clinic. The study included participants who gave birth at 37–42 weeks of pregnancy (term pregnancy), were aged over 18 years, and were able to converse and complete the questionnaires in Turkish. Those who had a chronic musculoskeletal disorder and history of orthopedic surgery that may be a reason of musculoskeletal symptoms other than pregnancy itself were excluded. The study protocol was approved by the local research ethics committee. The study adhered to the guidelines of the Declaration of Helsinki and informed consent was obtained from all participants.

### Procedures

The basic demographic and clinical characteristics of the participants including age, body mass index (BMI), weight gained during pregnancy, education level, occupation, parity, sex of infant, and exercise habits were collected from the medical charts and face-to-face interviews. A regular (at least twice a week) or irregular (not each week of the pregnancy) aerobic exercise of any kind including walking, jogging, swimming, biking, etc. irrespective of duration and intensity was questioned during pregnancy. The musculoskeletal complaints of the participants were questioned with interviews. Musculoskeletal pain sites were defined as hand–wrist, elbow, shoulder, neck, back, low back, hip, knee, and ankle–foot. The abovenamed body parts were shown to participants in a diagram of a human body in the questionnaire. Participants were asked if they had pain in those musculoskeletal sites during pregnancy.

In addition to the symptom of pain, the participants were also investigated in terms of some specific musculoskeletal symptoms including leg muscle cramps, carpal tunnel syndrome (CTS), meralgia paresthetica, and tarsal tunnel syndrome. The symptoms of all those musculoskeletal conditions were described to the participants. Leg muscle cramp was described as a strong, painful contraction or tightening of a muscle in the thigh, the calf or the foot that comes on suddenly and lasts from a few seconds to several minutes. The symptoms of CTS were described as tingling, numbness, weakness, or pain in the fingers or hand. Meralgia paresthetica was described as numbness, pain or an irritating sensation felt in the outer thigh. The symptoms of tarsal tunnel syndrome were described as tingling, numbness, or pain in the sole of the foot.

### Statistical analysis

Data analysis was performed using SPSS for Windows, version 15.0 (SPSS Inc., Chicago, IL, United States). The data were treated in a descriptive and inferential manner. The categorical variables were presented as absolute values and percentages, and the numerical variables as means and standard deviations. The chi-square test was used to compare the data. The statistical significance level was determined at *p* < 0.05.

## Results

A total of 184 female participants with a mean age of 30.9 ± 5.0 years participated in the study. Most of the participants had a university level of education (53.2%). The mean weight gained during pregnancy was 13.1 ± 4.8 kgs. Parity of the participants was mostly two or more (71.2%). The sex of the infants was male in 87 (47.3%) births and female in 97 (57.7%) births. Only 25 participants (13.6%) declared that they had undertaken a regular exercise program during pregnancy. All the demographic and clinical characteristics of the participants are presented in Table 1.

**Table 1.** Patient characteristics (*n* = 184).

	*n*	%
**Age (years)** ^*^	30.9 ± 5.0	
**BMI (kg/m^2^)** ^*^	24.0 ± 3.8	
**Weight gained during pregnancy (kg)** ^*^	13.1 ± 4.8	
**Education level**		
Primary	18	9.8
Secondary	11	6.0
High school	57	31.0
University	98	53.2
**Employed**		
Yes	68	36.9
No	116	63.1
**Parity**		
1	53	28.8
2	85	46.2
⩾3	46	25.0
**Sex of infant**		
Male	87	47.3
Female	97	57.7
**Exercise during pregnancy**		
Regular	25	13.6
Irregular	53	29.0
No	106	57.4

* Mean ± standard deviation.

BMI, body mass index.

**Figure 1.**
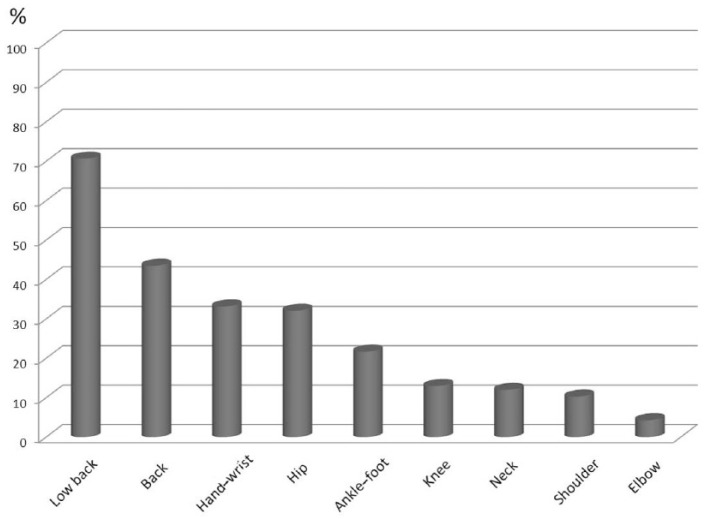
Distribution of pain sites through pregnancy.

The most frequent musculoskeletal complaints during pregnancy were low back pain (LBP) (*n* = 130, 70.7%), back (*n* = 80, 43.5%), hand–wrist (*n* = 61, 33.2%), and hip pain (*n* = 59, 32.1%) (Figure 1). The participants experienced musculoskeletal pain and symptoms most in the third trimester compared with the first and second trimesters, except for elbow, shoulder and neck pain (*p* < 0.05) (Table 2).

**Table 2.** Musculoskeletal pain sites of the participants (*n* = 184).

	First trimester *n* (%)	Second trimester *n* (%)	Third trimester *n* (%)
**Hand–wrist**	17 (9.2)	31 (16.8)^*^	54 (29.3)^$‡^
**Elbow**	2 (1.1)	7 (3.8)	5 (2.7)
**Shoulder**	11 (6.0)	14 (7.6)	15 (8.2)
**Neck**	13 (7.1)	16 (8.7)	20 (10.9)^$^
**Back**	32 (17.4)	42 (22.8)	75 (40.8)^$‡^
**Low back**	49 (26.6)	73 (39.7)^*^	123 (86.8)^$‡^
**Hip**	18 (9.8)	32 (17.4)^*^	55 (29.9)^$‡^
**Knee**	9 (4.9)	15 (8.2)	24 (13.0)^$‡^
**Ankle–foot**	12 (6.5)	17 (9.2)	36 (19.6)^$‡^

* Significant difference in comparison with first trimester.

$ Significant difference in comparison with first trimester.

‡ Significant difference in comparison with second trimester.

A total of 138 (75%) women suffered from leg muscle cramps during pregnancy. Leg muscle cramps were reported as occurring mostly at night-time (*n* = 106, 57.6%) and in the third trimester (*n* = 103, 55.9%) (Table 3). Peripheral neuropathies during pregnancy were reported as symptoms of CTS in 59 (32.1%), meralgia paresthetica in 41 (22.3%) and tarsal tunnel syndrome in 45 (24.5%) participants (Table 3).

**Table 3.** Specific musculoskeletal symptoms of the participants (*n* = 184).

	*n* (%)
**Leg muscle cramps**	
Presence of symptoms	138 (75.0)
**Time of symptoms**	
Daytime	26 (14.1)
Night-time	106 (57.6)
All day	6 (3.2)
**Period of the most severe symptoms**	
First trimester	3 (1.6)
Second trimester	32 (17.3)
Third trimester	103 (55.9)
**Carpal tunnel syndrome**	
Presence of symptoms	59 (32.1)
**Side of symptoms in the hands**	
Right	11 (5.9)
Left	6 (3.3)
Bilateral	42 (22.8)
**Meralgia paresthetica**	
**Presence of symptoms**	41 (22.3)
**Tarsal tunnel syndrome**	
**Presence of symptoms**	45 (24.5)

## Discussion

The results of this study framed a comprehensive analysis of the musculoskeletal disorders experienced during pregnancy. LBP was found to be the most frequent musculoskeletal complaint during pregnancy. The findings presented an increase in musculoskeletal symptoms in the third trimester. Various musculoskeletal problems may be an important source of discomfort during pregnancy.

Pregnancy causes considerable physiological effects on a woman’s body, affecting not only the cardiovascular, endocrine, and renal systems, but also the musculoskeletal system. Even though the musculoskeletal system can be affected at any time in pregnancy, this may be most prominent in the third trimester. The results of the present study showed there is a significant increase in hand–wrist, neck, back, low back, hip, knee, and ankle–foot pain in the third trimester compared with the other trimesters. It has been reported that postural and hormonal fluctuations, weight gain and fluid retention may account for increased musculoskeletal pain in the third trimester.^8^ More studies are needed to better understand the pathophysiology of musculoskeletal problems during pregnancy. It would enable the physician to make timely diagnoses and management.

LBP is very common during pregnancy and is estimated affecting 50–75% of pregnant women.^9^ In accordance, 70% of the pregnant women had LBP during pregnancy in the present study. LBP is a public health concern, because a large percentage of women who experience LBP during pregnancy continue to have pain after childbirth beyond the postpartum period.^10^ Therefore, it is important to prevent and treat LBP to prevent chronic pain and workforce loss. The reasons for LBP are manifold. It might be due to expected physiological changes, hormonal changes and the increase in body mass leading to altered compliance and increased mechanical stress on the spine. The enlarging gravid uterus, accompanying compensatory lumbar lordosis and shift of the center of gravity may increase strain on bones, muscles, ligaments of lumbar region. In addition, relaxed abdominal wall muscles, primarily the rectus abdominis, during pregnancy may not be able to maintain posture. As a compensatory effect, the paraspinal muscles are forced to undertake the whole function, become fatigued and thus, a cause of LBP.^9,10^

During pregnancy, the alteration of the mechanics requires the lower-extremity joints to adapt by absorbing extra force.^8^ Hip, knee, foot pain and leg spasms have been identified as the most common lower-extremity problems experienced during pregnancy. It has been shown that the hip is the most commonly affected area in the lower extremity. Vullo and colleagues^11^ reported that 34% of pregnant women experienced hip pain. Similarly, in the present study, 32% of women reported hip pain. Many pregnant women experience hip pain in their second or third trimester. It may be considered as a result of increase in mechanical load to hip joints in later stages of the pregnancy. However, some specific disorders should also be assessed. In a pregnant woman presenting with hip pain, transient osteoporosis of the hip or osteonecrosis of the femoral head must be considered. In addition, sacral fractures, acetabular labral tears, symphysis pubis diastasis or dysfunction, cauda equina syndrome, and sacroiliitis are rare causes of hip pain in pregnancy.^12–16^

Pregnancy-related CTS is the most frequent mononeuropathy during pregnancy. The incidence of CTS in pregnant women is two to three times higher than in women who are not pregnant. The rate of CTS in pregnancy varies from 0.23% to 62%.^17,18^ The findings of the current study were inconsistent with those of a study by Sapuan and colleagues,^19^ which showed that 25% of 333 postpartum patients had CTS during pregnancy and mostly with bilateral hand symptoms. CTS is often attributed to hormonal changes in pregnancy, with no clear etiology. Hormonal fluctuations, fluid accumulation with a tendency to edema, nerve hypersensitivity and glucose level fluctuations are factors that predispose pregnant women to CTS.^20^

There are a number of shortcomings related to the present study. Precise diagnostic labels such as imaging or clinical tests for individual painful condition was lacking. If the severity of pain was also assessed, it might give more value to the current study instead of only a yes/no for each pain disorder. This could have enriched the impact of these results and achieve the status of being a descriptive study. Since the study was conducted at a single university hospital, the sample may have been biased toward those with more complex medical conditions, thereby limiting the generalizability of these findings.

## Conclusion

Pregnant women face multiple musculoskeletal pain and symptoms, especially in the third trimester. LBP, back pain, hip pain and CTS are the most frequent painful conditions in pregnant women. The present study provides multiple entry points to further investigate the many musculoskeletal ailments reported, as well as the underlying physiological complexities associated with pregnancy.
